# Large field-of-view pine wilt disease tree detection based on improved YOLO v4 model with UAV images

**DOI:** 10.3389/fpls.2024.1381367

**Published:** 2024-06-20

**Authors:** Zhenbang Zhang, Chongyang Han, Xinrong Wang, Haoxin Li, Jie Li, Jinbin Zeng, Si Sun, Weibin Wu

**Affiliations:** ^1^ College of Engineering, South China Agricultural University, Guangzhou, China; ^2^ Guangdong Provincial Key Laboratory of Utilization and Conservation of Food and Medicinal Resources in Northern Region, Shaoguan University, Shaoguan, China; ^3^ College of Intelligent Engineering, Shaoguan University, Shaoguan, China; ^4^ College of Plant Protection, South China Agricultural University, Guangzhou, China; ^5^ College of Artificial Intelligence, Nankai University, Tianjin, China; ^6^ College of Forestry and Landscape Architecture, South China Agricultural University, Guangzhou, China

**Keywords:** pine wilt disease, UAV images, large field-of-view, deep learning, target detection

## Abstract

**Introduction:**

Pine wilt disease spreads rapidly, leading to the death of a large number of pine trees. Exploring the corresponding prevention and control measures for different stages of pine wilt disease is of great significance for its prevention and control.

**Methods:**

To address the issue of rapid detection of pine wilt in a large field of view, we used a drone to collect multiple sets of diseased tree samples at different times of the year, which made the model trained by deep learning more generalizable. This research improved the YOLO v4(You Only Look Once version 4) network for detecting pine wilt disease, and the channel attention mechanism module was used to improve the learning ability of the neural network.

**Results:**

The ablation experiment found that adding the attention mechanism SENet module combined with the self-designed feature enhancement module based on the feature pyramid had the best improvement effect, and the mAP of the improved model was 79.91%.

**Discussion:**

Comparing the improved YOLO v4 model with SSD, Faster RCNN, YOLO v3, and YOLO v5, it was found that the mAP of the improved YOLO v4 model was significantly higher than the other four models, which provided an efficient solution for intelligent diagnosis of pine wood nematode disease. The improved YOLO v4 model enables precise location and identification of pine wilt trees under changing light conditions. Deployment of the model on a UAV enables large-scale detection of pine wilt disease and helps to solve the challenges of rapid detection and prevention of pine wilt disease.

## 1 Introduction

Pine wilt disease (PWD) is caused by pine wood nematode (PWN), which is known for its high destructiveness (Kobayashi et al., 2003). The disease has been widely distributed in Asia, especially in China, Japan, and South Korea, where it has caused the most damage (Kikuchi et al., 2011). The spread of PWD is swift. Once a diseased tree is found, nearby pine trees may also be infected (Asai and Futai, 2011). PWNS feed on and infest pine trees, causing the trees to weaken and die (Yun et al., 2012), resulting in losses to forestry production and the ecological environment. Countries have strengthened quarantine and control measures to cope with the spread of PWD. The spread of PWD poses a threat to Asia’s forestry and ecological environment (Wu et al., 2020). Therefore, monitoring PWD is of great significance for the safety of China’s forest resources (Schröder et al., 2010). The application of drone remote sensing technology has dramatically improved the efficiency of forest resource surveys (Kentsch et al., 2020). Traditional monitoring techniques rely on low-level semantic features extracted from remote sensing images, making them susceptible to factors such as noise, lighting, and seasons, which limits their application in complex real-world scenarios (Park et al., 2016). Using drones to aerially photograph areas affected by PWD, the location and degree of diseased trees can be visually observed from the aerial images, and targeted measures can be taken to deal with diseased trees, reducing the workload of manual investigations. It is of great significance to use drones combined with artificial intelligence algorithms to detect pine wilt disease, which significantly improves the detection efficiency of pine wilt disease.

With the rapid development of drone monitoring technology and image processing technology, drone remote sensing monitoring methods have gradually been applied in PWD monitoring (Syifa et al., 2020; Vicente et al., 2012). When drones are used to aerially photograph areas affected by pine wilt disease, visible light cameras are carried to obtain ground images within the scope of the PWD epidemic, and the images are transmitted to the display terminal for automatic identification and positioning of diseased trees by the trained target detection algorithm (Kuroda, 2010). The use of drones for automatic monitoring of PWD can improve the efficiency of diseased tree monitoring. Compared with satellite remote sensing monitoring, drone remote sensing monitoring has a lower cost and more straightforward operation. Applying this technology in PWD detection is beneficial to the protection of pine tree resources and the stability of the ecological environment (Gao et al., 2015; Tang and Shao, 2015).

In target detection, accurate feature extraction from images is a crucial issue affecting model performance. Traditional image target detection uses machine learning algorithms to extract image features. However, because machine learning algorithms can only extract shallow feature information from images, the performance of target detection is challenging to improve (Khan et al., 2021). Machine learning algorithms use manually designed feature operators to extract feature vectors of targets in the image, and based on these feature vectors, use statistical learning methods to achieve intelligent visual detection of image targets (Tian and Daigle, 2019). These algorithms rely on colors or specific shapes whose features are not stable enough, resulting in detection mode. Thus, the adaptability and robustness of the model to the environment are not good enough (Long et al., 2015). Therefore, deep learning algorithms have emerged (Li et al., 2023), and it has been successfully applied in fields such as computer vision, speech recognition, and medical image analysis. This algorithm uses convolutional neural networks to extract image features, which can extract deep-level feature information of image targets, thereby improving the detection accuracy of diseased trees (Lifkooee et al., 2018). The theoretical system of target detection algorithms has gradually improved as research in this subject has progressed, and many distinct method frameworks have been employed in many image detection fields (Zhang and Zhang, 2019). Li proposes a multi-block SSD method based on small object detection to the railway scene of UAV surveillance (Li et al., 2020). Xu extends the Faster RCNN vehicle detection framework from low-altitude drone images captured at signalized intersections (Xu et al., 2017). The focus of the research is how to change the structure of the algorithm model and achieve a balance between detection accuracy and processing time (Hosang et al., 2016).

Under changing lighting conditions, the texture features of the image change, resulting in a decrease in detection accuracy (Barnich and Van, 2011). There are relatively few algorithms for monitoring pine wilt diseased trees in the lighting change scene, and most of the target detection algorithms for diseased trees have complex structures, low detection accuracy, and low computational efficiency (Zhang et al., 2019). Huang et al. Constructed a densely connected convolutional networks (D-CNN) sample dataset, using GF-1 and GF-2 remote sensing images of areas with PWD. Then, the “microarchitecture combined with micromodules for joint tuning and improvement” strategy was used to improve the five popular model structures (Huang et al., 2022). In 2021, a spatiotemporal change detection method to improve accurate detections in tree-scale PWD monitoring was proposed by Zhang et al., which represents the capture of spectral, temporal, and spatial features (Zhang et al., 2021).

Currently, most of the detections for pine wilt are done by biological sampling, which is time-consuming and labor-intensive. Research on the detection of pine wilt disease using unmanned aerial vehicle (UAV) has mainly focused on stable light conditions, and little attention has been paid to the detection of pine wilt disease under changing light conditions, resulting in the low detection accuracy of the existing models, as well as the inability of their improved methods to detect disease spots under changing light conditions. And there is the problem of small field of view and small number of targets. The research object of this paper is PWD tree, by increasing the flying height of UAV, increasing the field of view range of the camera, increasing the number of image targets, and based on this, a set of algorithms for detecting and recognizing the targets of diseased tree is proposed, which provides theoretical and practical support for detecting and recognizing the targets of remote sensing images by UAV.

In conclusion, this paper proposes a YOLO v4 target recognition algorithm based on the Attention Mechanism Module to establish a model for rapid localization and accurate recognition of pine nematode disease trees under dynamic light changes. Further, combining it with UAV image technology realizes rapid multi-target detection over a large field of view. This can save time in investigating pine wood nematode disease and realize prevention in advance, which is of great significance for preventing the spread of pine wood nematode disease.

## 2 Experimental parameters and YOLO v4 network structure

### 2.1 Sample collection sites and UAV images acquisition

**Figure 1:**
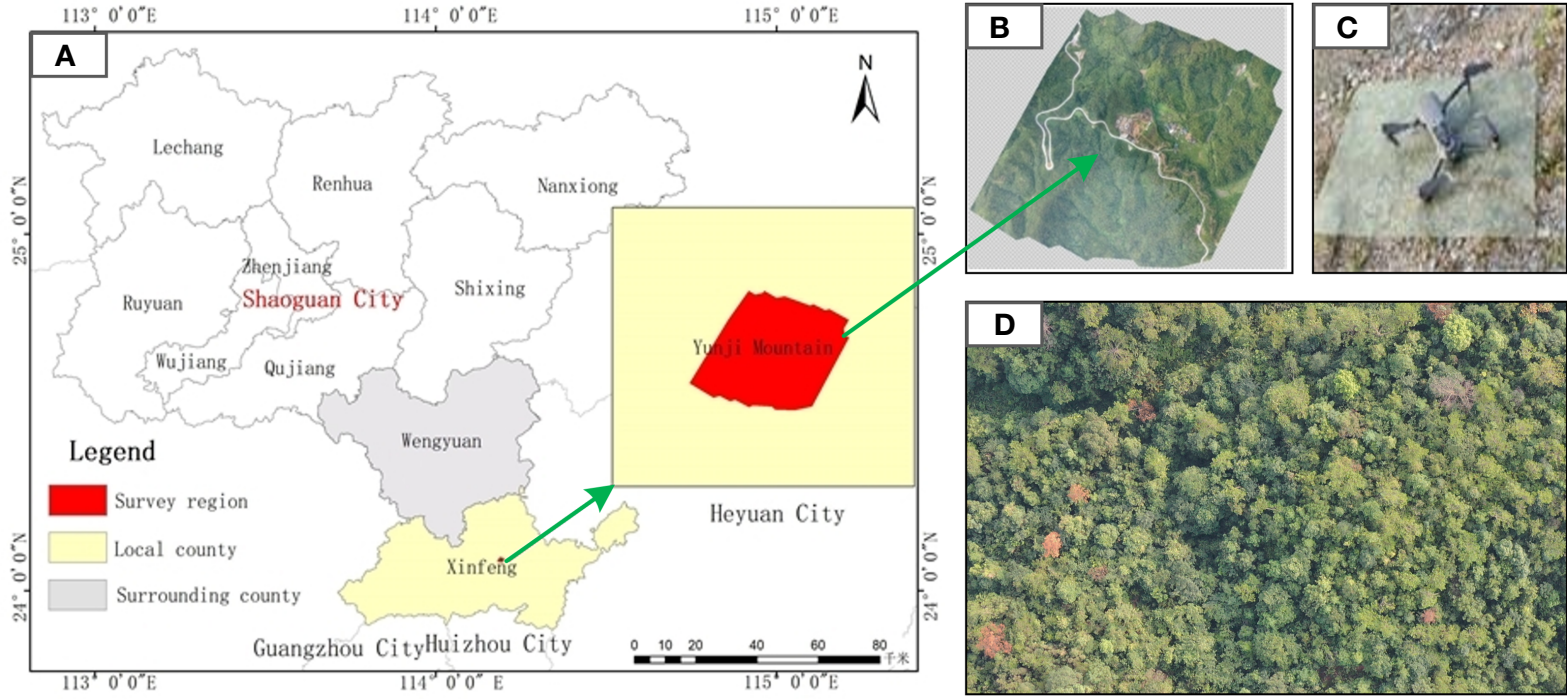
Geographical location diagram of UAV images acquisition. **(A)** Geographical location map of the research area **(B)** UAV orthophoto map **(C)** Drone appearance diagram **(D)** Single UAV aerial photo.

The prominent peak of Yunji Mountain has an elevation of 1434.2 meters and is located at 24°07’ north latitude and 114°08’ east longitude (**Figure 1A**). It is located in the north of Guangzhou City, in the central part of Xinfeng County, 10 kilometers away from the county town. It belongs to the natural ecosystem transitional zone from the South sub-tropical zone to the Central subtropical zone, with a jurisdictional area of 2700 hectares. The panoramic image collected by the drone was taken in multiple shots and stitched together to form a complete image. The collection area includes a winding road and houses distributed along the roadside. The mountain is higher in the northeast and lower in the southwest as shown in **Figure 1B**.

The visible light images were acquired using the DJI Mavic 2 drone, equipped with ten sensors distributed in six directions: front, rear, left, right, up, and down. The sensor model is 1-inch Complementary Metal Oxide Semic (CMOS), and the captured image resolution is 5472×3684. The drone can reduce air resistance by 19% during high-speed flight, and its maximum flight speed can reach 72 km/h, with a flight time of up to 31 minutes, the experimental drone is shown in **Figure 1C**.

The illumination can affect the clarity of the drone remote sensing image collection. Due to the continuously changing natural lighting conditions over time and weather, the lighting conditions greatly affect the image quality, resulting in complex information in the collected images of diseased trees. According to the lighting conditions of the photos, they can be divided into two categories: sufficient light and insufficient light. The light intensity was measured by an illuminometer.

To balance the image quality and the diseased tree target detection network, all remote sensing images of diseased trees are uniformly resized to a resolution of 416×416 pixels. The uneven lighting caused by changes in the lighting conditions affects the quality of the images (**Figure 1D**). The change in the lighting environment poses a significant challenge for object detection. Compared with the photos collected under sufficient lighting conditions, whose illuminance is 10826 lux, the remote sensing images of diseased trees collected under insufficient lighting conditions contain a large amount of noise. The visibility of objects such as diseased trees, houses, and roads is poor, resulting in blurred targets and severe distortion of details (Zuky et al., 2013).

### 2.2 Experimental environment configuration and training parameter settings

The YOLO v4 and its improved diseased tree detection algorithm run on the Windows 10.0 system with 32 GB of memory. This experiment uses an NVIDIA GeForce RTX 3080 Ti graphics card with 12 GB of memory and an 8-core 11th Gen Intel Core i7–11700KF CPU. The central frequency of the CPU is 3.6 GHz. Adopting an object detection algorithm based on PyTorch, the code runs in Python 3.7 environment. The object detection network is built using the Python language. In addition, third-party library packages such as numpy, opencv, and panda. Pytorch are Python-based machine learning libraries that can achieve powerful GPU acceleration.

The model parameters of YOLO v4 are set as shown (**Table 1**). The input image size is 416×416, the optimizer uses Adam, a total of 50 epochs are trained, the threshold of the prior box is set to 0.5, and the loss function is cross-entropy loss. The model’s training process is divided into two stages: frozen training and unfrozen training (Liu et al., 2020). During the firm training process, the pretraining weights of the backbone network do not need to be trained, which can improve the training efficiency of the networks, and addicts were also used. Usually, an increase in detection accuracy leads to an increase in the complexity of the model, but due to the limitations of computer arithmetic thus leading to slow computation. Therefore, the use of higher computing power computers or multi-CPU parallel computing can improve the detection time and accuracy, but it is a challenge to balance the model size and cost control.

**Table 1:** Model training parameter settings.

Parameter Name	Freeze Training Phase	Unfreezing Training Stage
Epoch	1–25	25–50
Learning rate	0.001	0.0001
Batch size	4	4

### 2.3 YOLO v4 network structure and detection process

YOLO v4 is an improvement on YOLO v3, retaining most of the structure of the YOLO v3. The improved parts of the network architecture include the input part, the leading feature extraction network, the neck network, and the head network (Bochkovskiy et al., 2020). Unlike YOLO v3, the feature extraction network of YOLO v4 is replaced by CSPDarknet53. The main feature extraction network comprises CSPDarknet53, and Cross Stage Partial (CSP) can effectively enhance the feature extraction ability of the convolutional network (Hui et al., 2021; Deng et al., 2022). The feature extraction network used by YOLO v4 is CSPDarknet, composed of the CSPX and CBM modules arranged alternately (Jiang et al., 2013). The structure of CSPX is shown in (Acharya, 2014; Fan et al., 2022).

**Figure 2:**
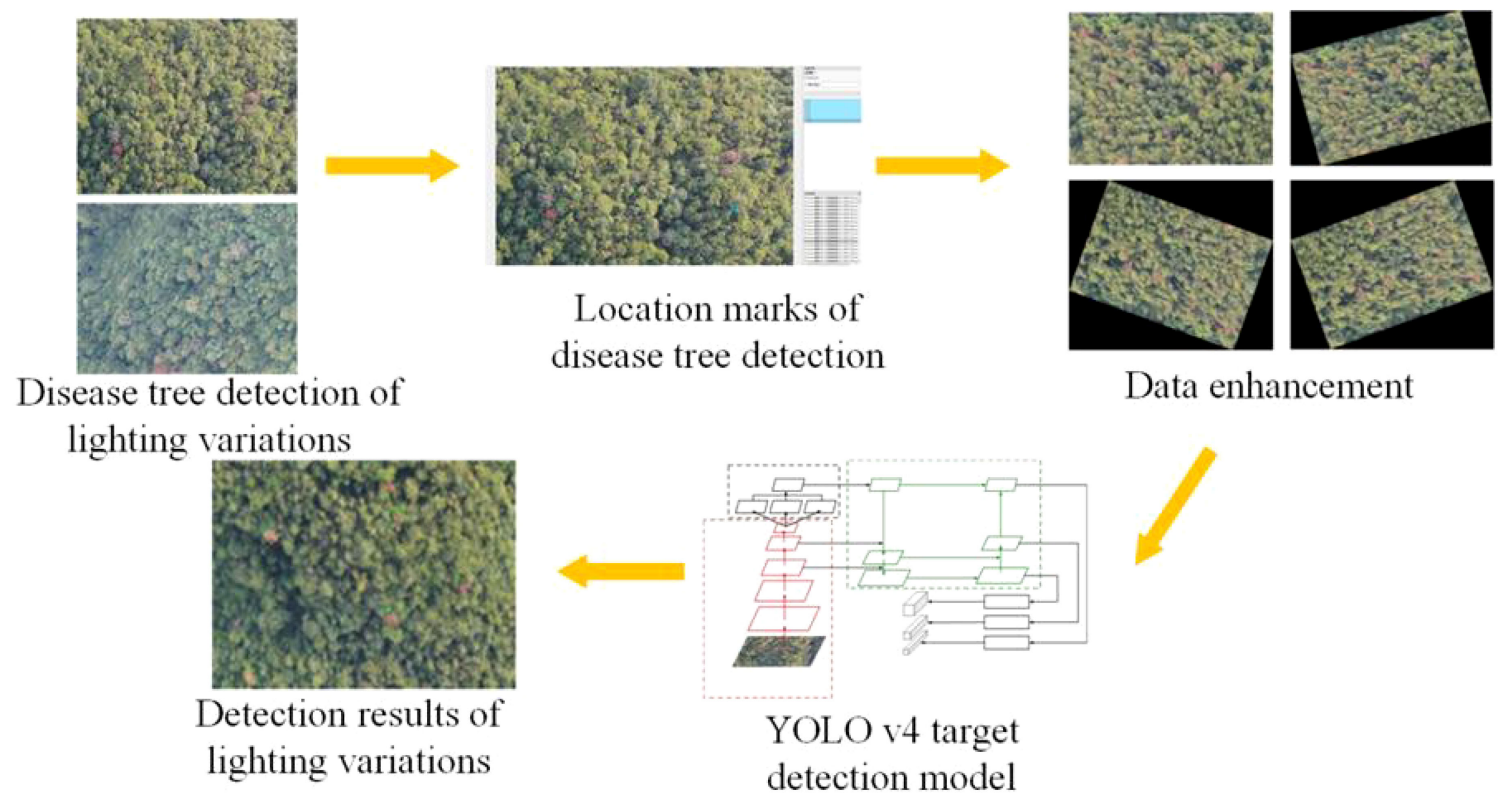
Disease tree target detection process for YOLO v4.

First, visible light images of PWD trees collected by drones are annotated with the Labeling tool to save the detection box position and category information as an XML file. The training set images are rotated at different angles and input into YOLO v4 for training to increase the diversity of training samples. The trained model outputs detection boxes for the test set images (**Figure 2**).

In order to increase the detection accuracy of the model, this study modified the structure of the YOLO v4 model. By embedding attention mechanism and feature enhancement module in the YOLO v4 model improves the model’s feature extraction ability. Determine the optimal model structure through ablation experiments.

## 3 Model improvement and methodology

### 3.1 Data enhancement and attention mechanism test

**Figure 3:**
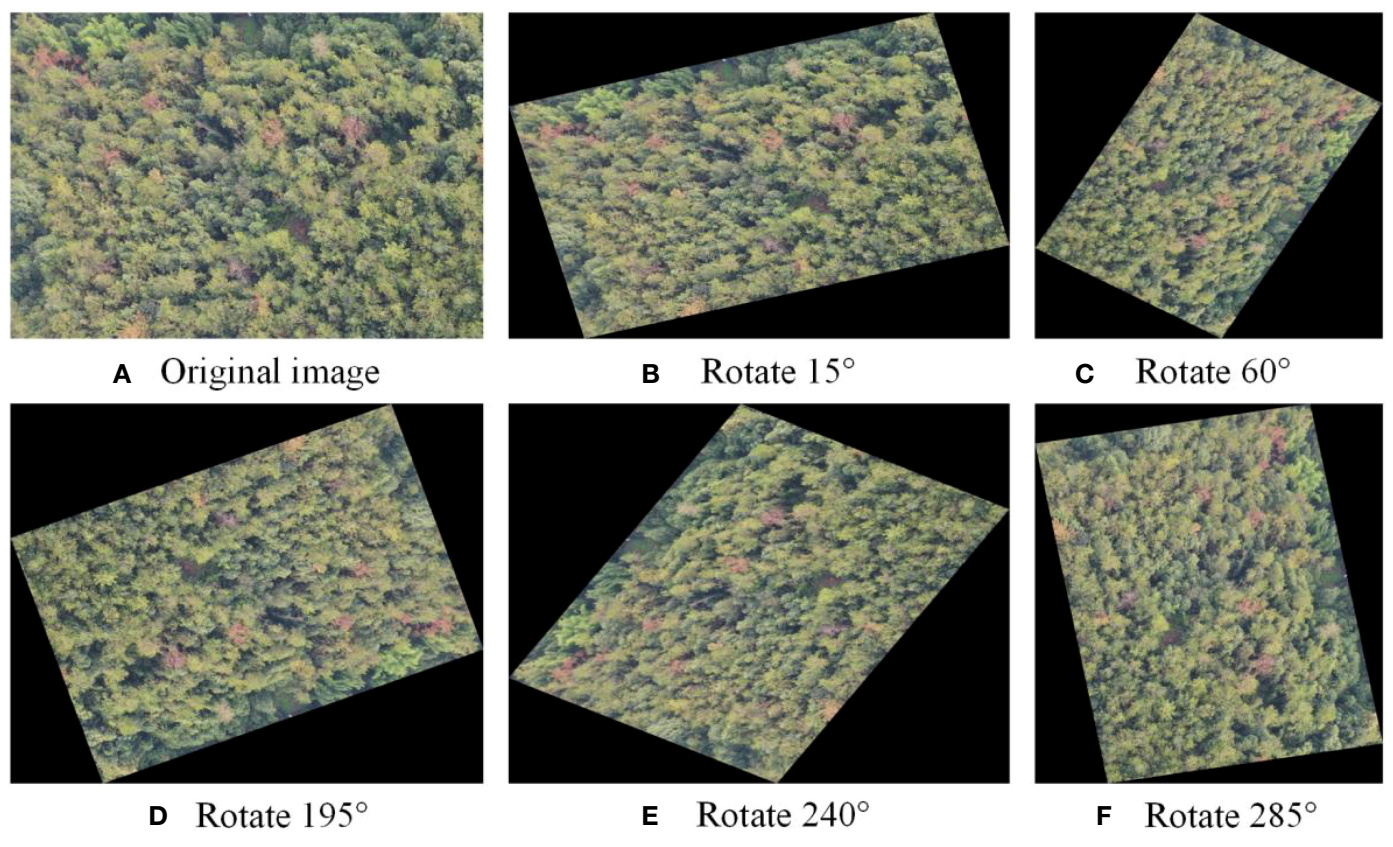
The diagram of data enhancement. **(A)** Original image **(B)** Rotate 15° **(C)** Rotate 60° **(D)** Rotate 195° **(E)** Rotate 240° **(F)** Rotate 285°.

To increase the diversity of training samples, prevent over fitting during model training, and improve the accuracy of model detection. A widespread way to enhance image data is to perform geometric transformation, such as cropping, rotating, translating, and adjusting the image’s brightness (Kim and Seo, 2018). This study used the rotation method to perform data augmentation on the training set samples. Five different angles, 15°, 60°, 195°, 240°, and 285°, were used to rotate the training set images, corresponding to **Figures 3B–F**, respectively. And the original image is showed in **Fiqure 3A**.

Convolutional neural networks contain the invariance property, which allows the network to preserve invariance to images under changing illuminations, sizes, and views. As a result, by rotating the acquired drone diseased tree photographs from various angles, the neural network will recognize these images as distinct (Moeskops et al., 2016). Due to the limited number of diseased tree images, a large sample set was added by augmenting the images through rotation at different angles. Five different angles were used to rotate the images, and five different images were obtained. The schematic diagram of the diseased tree images before and after sample augmentation is shown in the figure, and the number of images obtained after image transformation reached 7218, with 515 images in the test set. The above method was used to augment the sample data in the training set. The initial data in the training set was 1203 images, which was expanded six-fold. After rotating the images, the sample data set was expanded, and the expanded data was divided into a training set and a validation set. The training set contains 5052 images, the validation set contains 2166 images, and the test set contains 515 images.

The recognition results on the diseased pine tree dataset are compared (**Table 2**). It can be seen from the table that before data augmentation, the mean average precision (mAP) of the diseased pine tree detection was 77.45%. After data augmentation, the detection accuracy of the diseased pine tree was slightly improved, with an mAP of 77.81%, an increase of 0.36%. The accuracy increased by 0.22%, the specificity increased by 0.01, the recall increased by 2.22%, and precision decreased slightly. Overall, the detection accuracy of the diseased pine tree was improved. Data analysis shows that data augmentation can improve the detection effect of the diseased pine tree.

**Table 2:** Data enhancement effect.

	mAP/%	F1	Accuracy/%	Precision/%	Recall/%
Before augmentation	77.45	0.73	84.91	83.38	65.25
After augmentation	77.81	0.74	85.13	81.74	67.47

### 3.2 Attention mechanism addition position test

**Figure 4:**
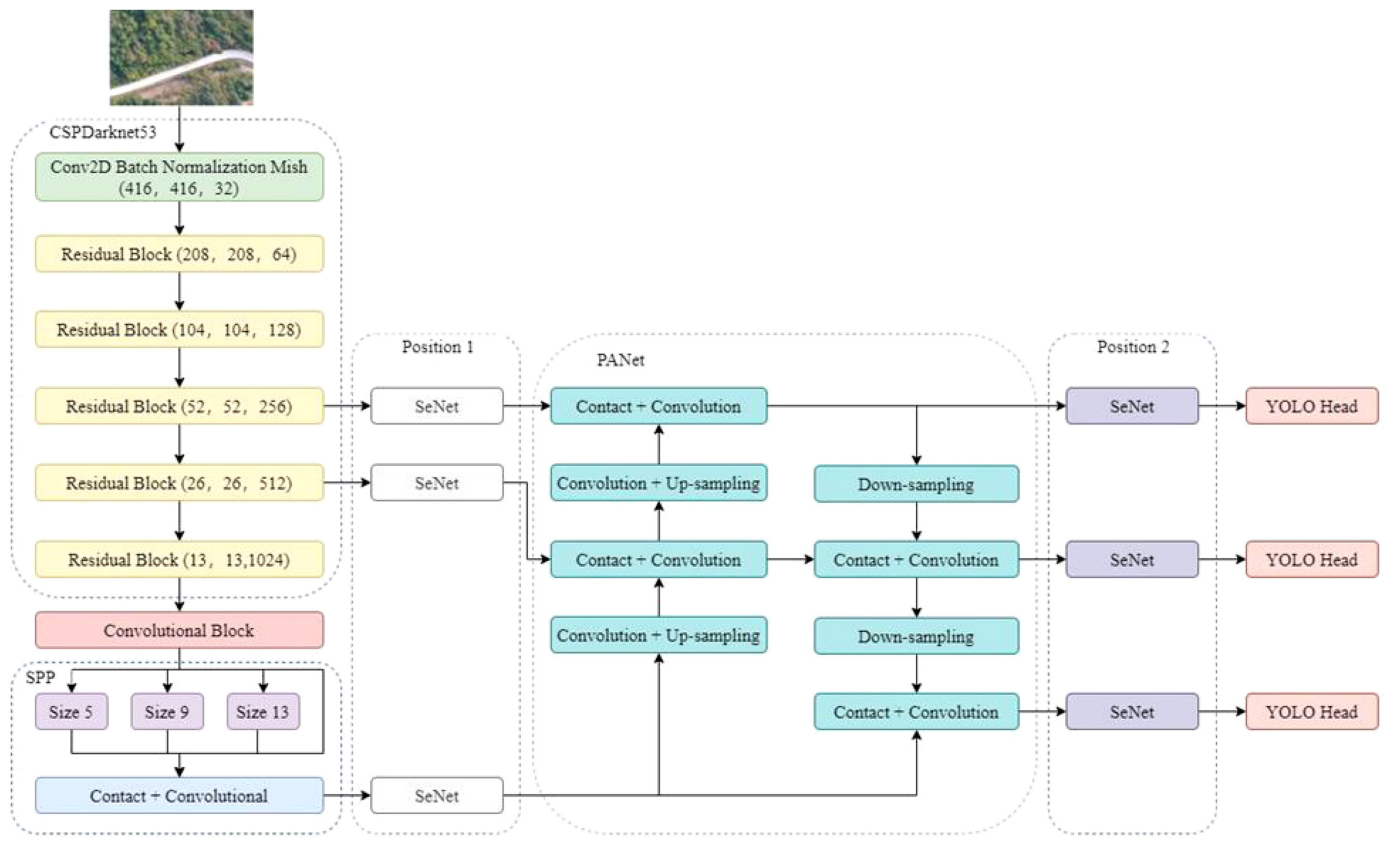
Different locations for adding attention mechanisms.

To determine the appropriate position for adding the attention mechanism, the detection performance of two different positions with the attention mechanism added in the YOLO v4 network structure was compared. Position 1 added the attention mechanism after the last three feature layers of the backbone feature network, before the feature pyramid network. In contract, position 2 added the attention mechanism before the three YOLO detection heads (**Figure 4**).

The detection accuracy of the attention mechanism at different positions is shown in **Table 3**. When the Squeeze-and-Excitation Networks (SENet) attention mechanism was added at position 1, the mAP of the test set was 79.29%. When the SENet attention mechanism was added at position 2, the mAP of the test set was 78.09%. The accuracy and recall in position 1 were higher than in position 2, with an increase of 0.42% and 1.76%, respectively, indicating that adding the attention mechanism at position 1 achieved higher detection accuracy and better detection performance.

**Table 3:** Evaluation indicators for detection accuracy of different addition positions in attention mechanisms.

	mAP/%	Accuracy/%	Precision/%	Recall/%	F1	FPS/(sheets/s)
Position 1	79.29	91.57	83.74	69.95	0.76	49.78
Position 2	78.09	91.15	83.84	67.19	0.75	50.24

**Figure 5:**
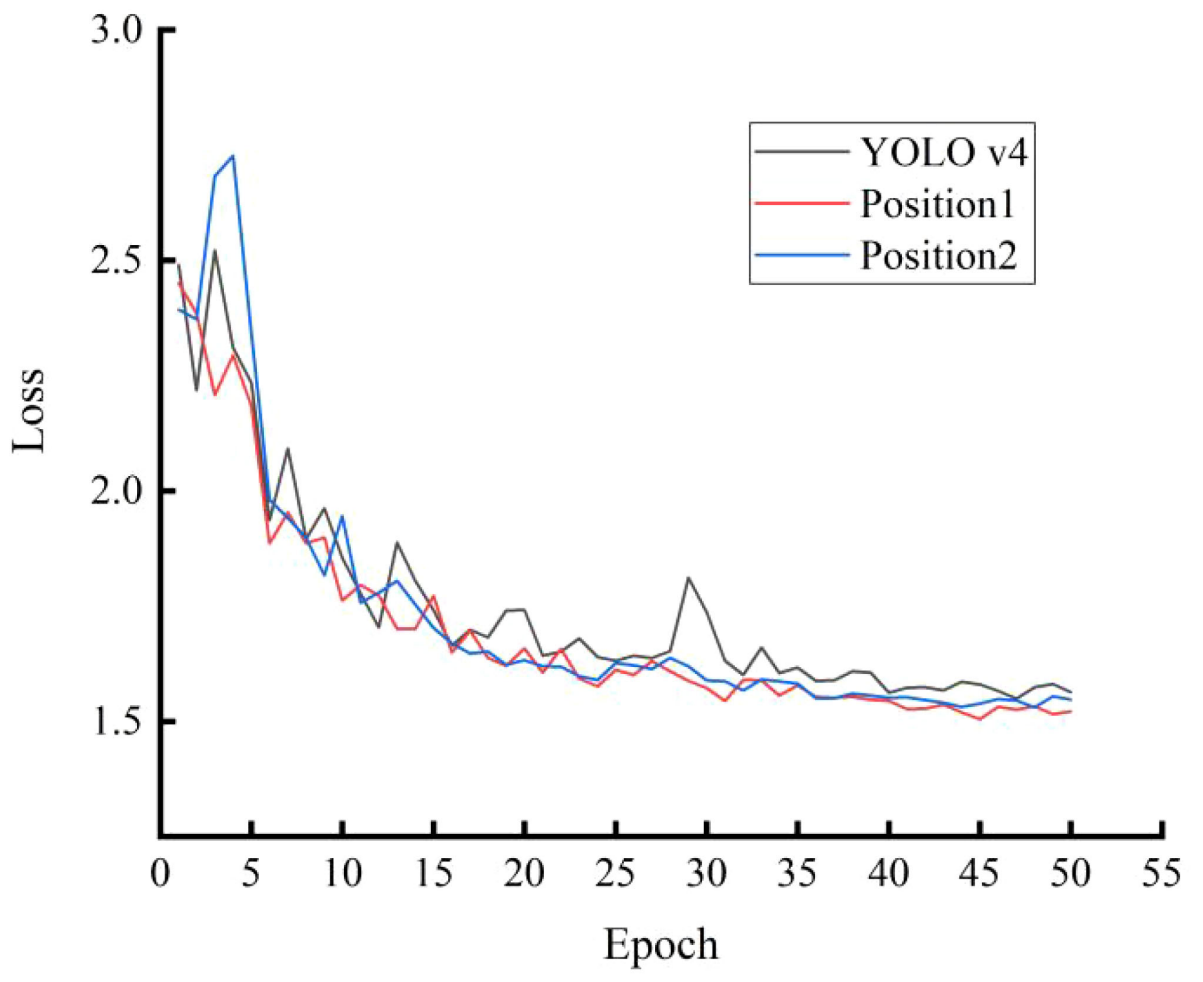
Loss curve of test set with different addition positions in attention mechanisms.

**Figure 5** shows the loss curves of the attention mechanism SENet at different embedding positions. The loss curves indicate that all three models can converge quickly during training. The loss in the test set decreases rapidly before 20 epochs and slows down when trained to 40 epochs. After 40 epochs, the loss value tends to stabilize. However, the loss curve of the YOLO v4 model fluctuates more. After convergence, the model with attention mechanism SENet embedded in position 1 has a lower loss value. Therefore, the feature extraction effect of the attention mechanism SENet embedded in position 1 is better.

### 3.3 Attention mechanism type test

**Figure 6:**
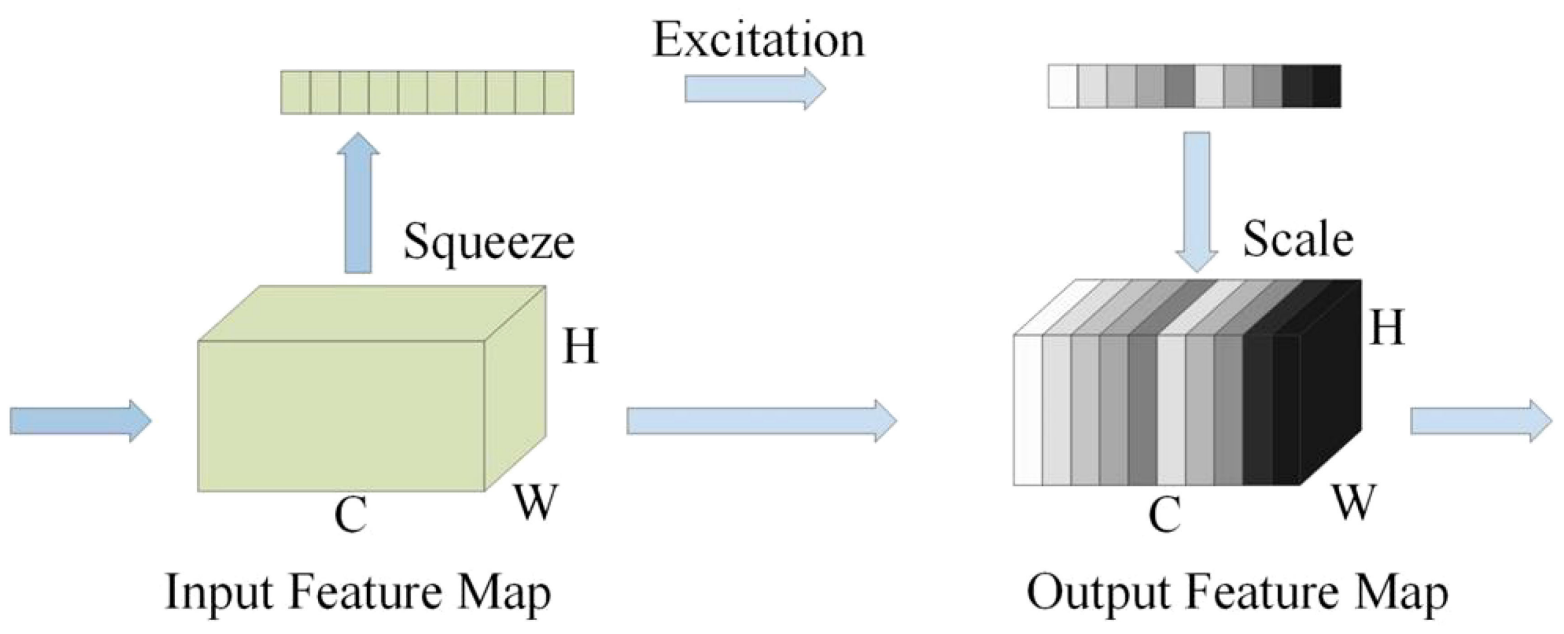
SENet channel attention mechanism.

Channel attention module SENet includes squeeze, excitation, and weight calibration operations (Hu et al., 2018). The channel attention module SENet can learn feature weights based on the loss function and then re-calculate the weights for each feature channel so that the object detection model places more attention on the features, thereby improving the object detection accuracy (**Figure 6**).

The information propagation in the network structure follows the order of input feature map, global pooling layer, feature matrix with a size of 1×1×C, one-dimensional convolution structure with a convolution kernel size of k, and output feature map. The forward propagation process outputs channel weight parameters, which are then loaded into the input feature matrix using matrix multiplication. The core idea of efficient channel attention network (ECA-Net) is to introduce channel attention after the convolutional layer to dynamically adjust the response of different channels (Xue et al., 2022).

**Figure 7:**
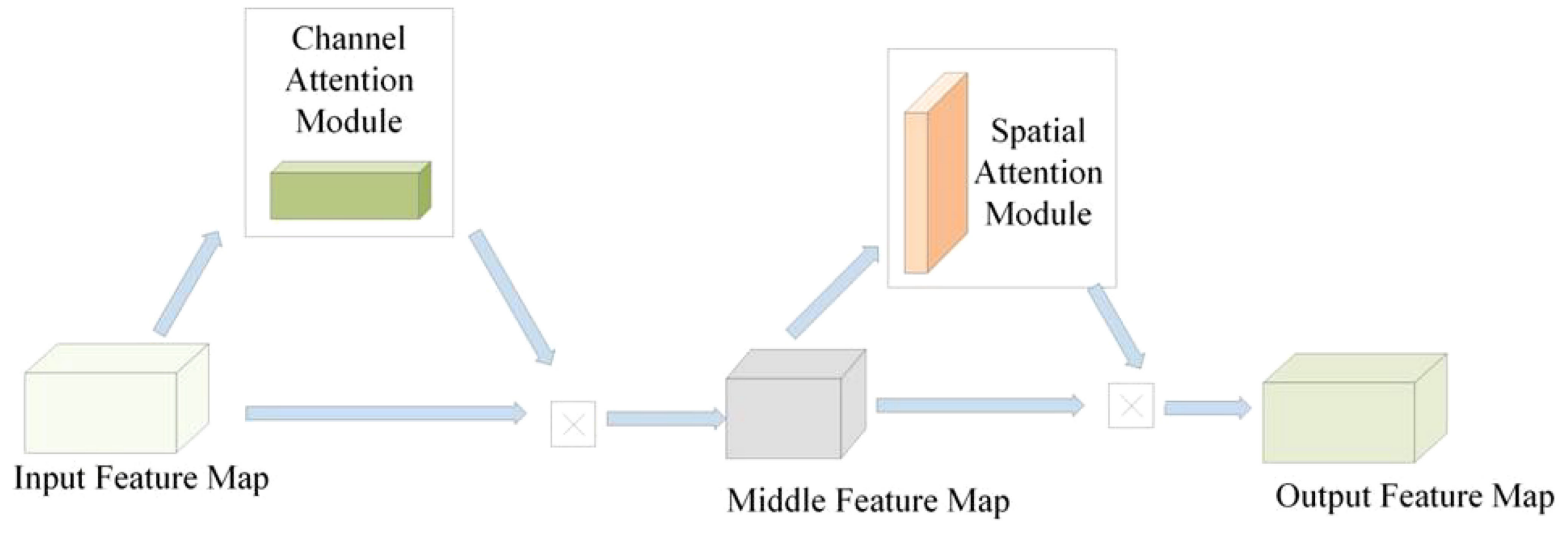
Schematic diagram of CBAM module.

The convolutional block attention module (CBAM) feature module is composed of a channel attention feature module and a spatial attention feature module (Woo et al., 2018). The channel attention feature module performs global max pooling and global average pooling operations on the input feature map to obtain two feature maps, which are then input into a multi-layer perceptron network (Selvaraju et al., 2020). The multi-layer perceptron network sums the two feature maps obtained and inputs them into a sigmoid activation function to obtain the channel attention feature weights (**Figure 7**). Finally, the weights are multiplied by the input feature map to obtain the intermediate feature map.

**Figure 8:**
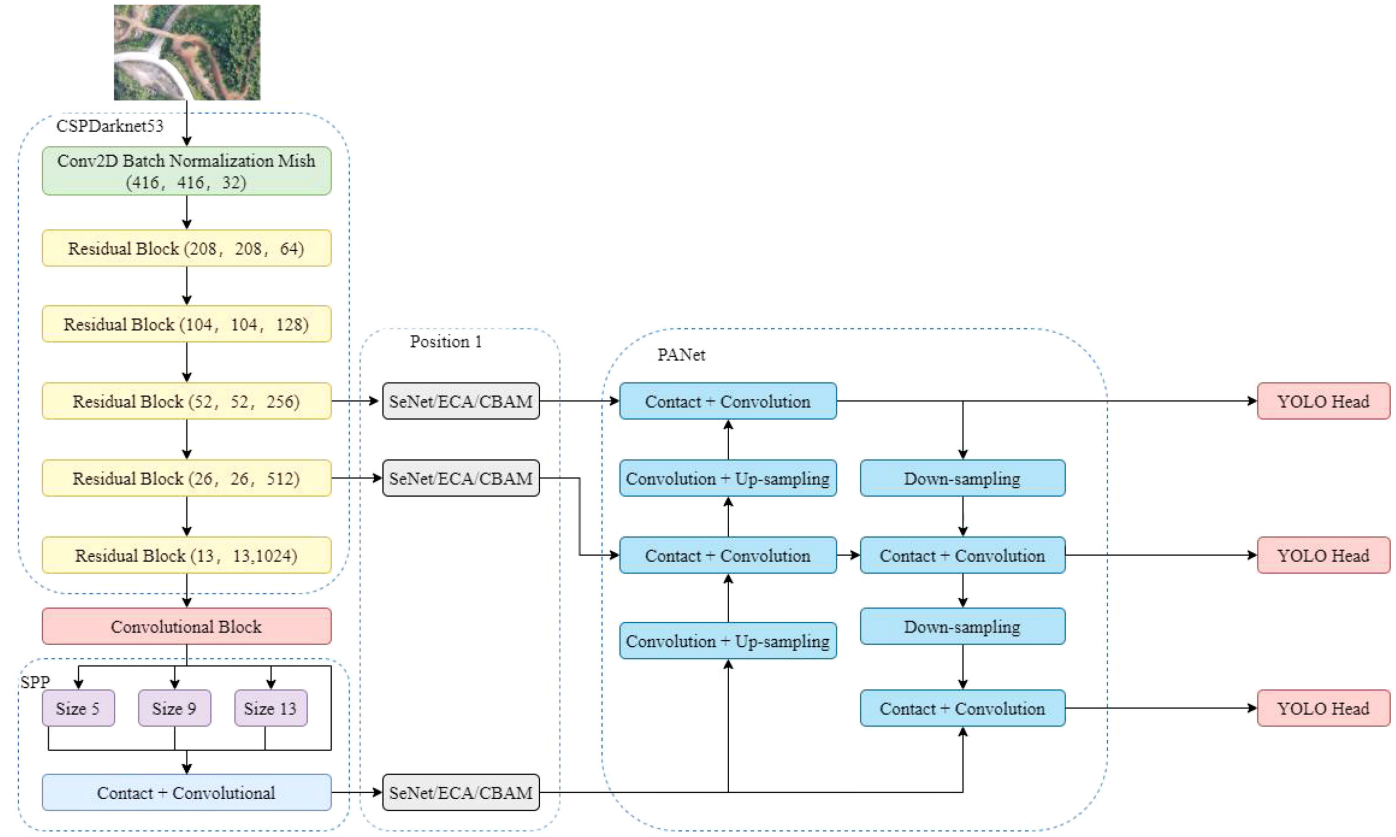
The addition positions of different attention mechanisms.

To improve the accuracy of the YOLO v4 object detection model, this work introduced three attention mechanisms to the feature pyramid of the YOLO v4 model for feature extraction. Three types of attention mechanisms include SENet, ECA and CBAM (**Figure 8**).

The accuracy and detection speed of the model before and after improvement were tested in **Table 4**.

**Table 4:** Evaluation of detection accuracy for different attention mechanisms.

	mAP/%	Accuracy/%	Precision/%	Recall/%	FPS/(sheets/s)
YOLO v4	77.81	85.13	83.38	65.25	52.53
ECA-YOLO v4	79.00	91.33	83.75	68.26	51.98
SENet-YOLO v4	79.29	91.57	83.74	69.95	49.78
CBAM-YOLO v4	79.07	91.15	85.22	65.91	53.1

The mAP of the YOLO v4 model on the test set was 77.81%, with a recall of 65.25%, precision of 83.38%, and accuracy of 85.13%. After adding attention mechanisms, the detection accuracy of the model was improved to varying degrees. Among them, the addition of the SENet attention mechanism achieved the most significant improvement in detection accuracy, with an increase in mAP from 77.81% to 79.29%, an increase of 1.48% compared to the YOLO v4 model, and an increase in accuracy from 85.13% to 91.57%. FPS was used to assess the running speed of the four models. The running speed of the YOLO v4 model was 52.53 frames per second (fps), while the speed of the SENet-YOLO v4 model was slower, with an FPS of 49.78, a decrease of 2.75 fps compared to the original YOLO v4 model, indicating that the processing speed of the model decreased after adding SENet. Although the running speed of the model decreased, the added SENet showed an accuracy improvement of over 1% on the diseased pine tree dataset, indicating the effectiveness of the model improvement. Based on the evaluation of the four models’ test accuracy and speed, the SENet-YOLO v4 model had the best testing performance. The accuracy of this model was the best, with an mAP of 79.29% on the test set, an increase of 1.48% compared to the YOLO v4 model. At the same time, among the four models, the CBAM-YOLO v4 model had the fastest processing speed, with an FPS of 57.32 on the test set, an increase of 0.9 fps compared to the YOLO v4 model. These show that the YOLO v4 model embedded with the SENet module can extract target features in more detail, which is beneficial for target classification. Although the detection speed decreased, the test accuracy was improved, and the model performance was optimized.

## 4 Model improvement and methodology

### 4.1 Ablation test

Three groups of ablation experiments were conducted to demonstrate the effectiveness of each improvement method used in the YOLO v4 network, including feature enhancement modules, feature fusion modules, and attention mechanisms. All parameters except for the testing module were kept consistent during the ablation experiments.

**Figure 9:**
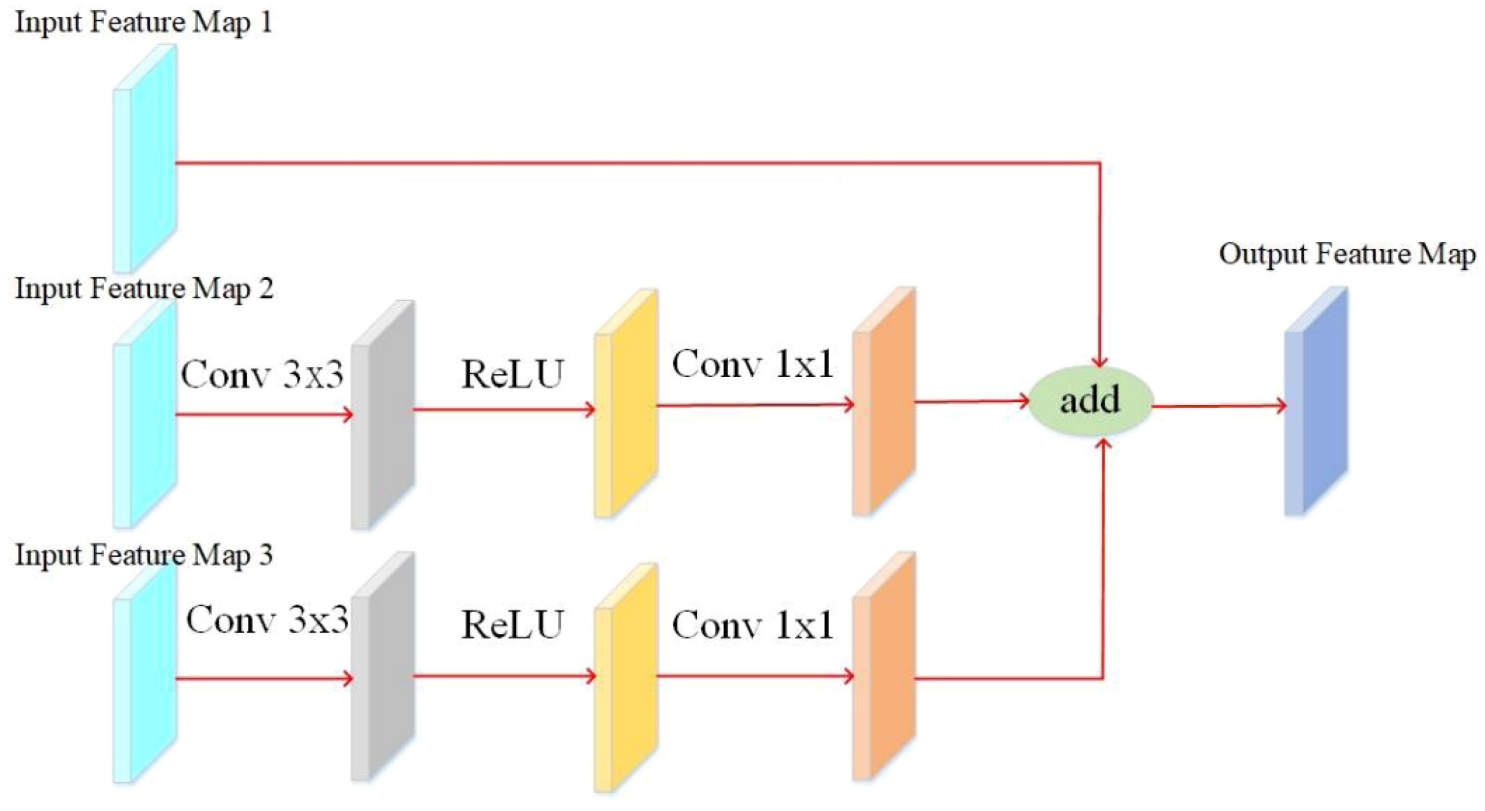
Feature enhancement module.

As different layers contain significantly different information, it is necessary to improve the adaptability of the feature layers to the target and the stability of the model for targets of different sizes. The working principle of this module is to perform three different operations on the input feature map (**Figure 9**). The second operation uses a 3x3 convolution operation, followed by the ReLU activation function, and ends with a 1x1 convolution operation. The third operation is the same as the second operation but with different padding for the 3x3 convolution. The three operations are then combined, and the enhanced feature map is output to improve the network’s feature extraction ability further and acquire adequate information about the target in the feature map, acting as a feature enhancement (Liang et al., 2021).

**Figure 10:**
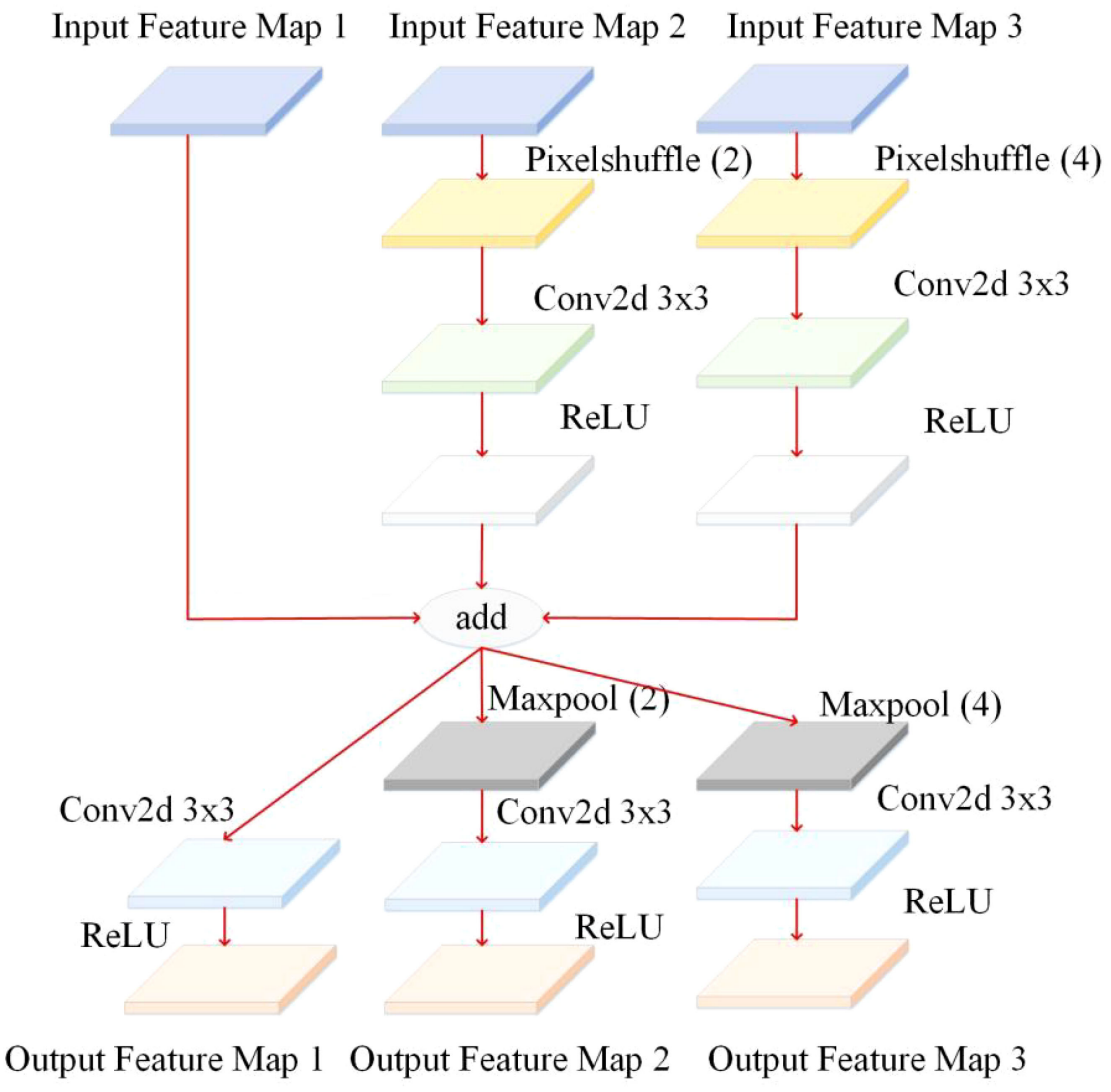
Feature fusion module.

In the YOLO v4 backbone feature extraction network, there are differences in the information contained in the feature maps of different layers (Sun et al., 2021). Deep feature maps contain rich semantic information, but small targets have less information and are usually used to detect large targets. Low-feature maps contain much detailed information but lack rich semantic information for detecting small targets. In order to better extract the feature information of diseased pine trees, a feature fusion module is designed, as shown in the **Figure 9**. This module adds three layers of feature maps to obtain the context information of diseased pine trees fully and then adds the outputs of three branches to achieve feature fusion (Sun et al., 2005). Three different scales of the backbone feature extraction network in the YOLO v4 model. The working principle of this module is: three feature maps of different sizes are used as inputs for the three branches, and the input feature maps of the middle branch are enlarged to adjust the size of the feature maps, and then 3×3 to extract the features of the input feature map, and finally use the Activation function rectified linear unit (ReLU). The operation process of the input feature map for branch 3 is the same as that for branch 2. Due to the difference in size between the input feature maps of the third branch and the input feature maps of the second branch, there is a difference in magnification between the input feature maps of the third branch and the second branch. The feature maps are processed by the first branch, and the other two branches are added and fused. The fused feature map is further divided into three branches for processing, and the feature map of the first branch is processed through three steps. After the convolution operation of 3×3, use the Activation function ReLU to process, and output the feature map (**Figure 10**). The difference between the other two branches is that before activating the operation, the maximum pooling operation is used to adjust the size of the feature map to match the input feature map size of the corresponding branch. By fusing feature maps from adjacent layers through the feature fusion module, the semantic differences between different feature channel layers are further reduced. This module can be used to collect contextual information of different scales and improve detection accuracy (Wu et al., 2021).

The effectiveness of the target detection network improvement methods was evaluated using the mAP evaluation metric, and the impact of each module on the overall network performance was analyzed. The “√” in the table indicates that the corresponding module was added to the original YOLO v4 network, while the absence of “√” indicates that the corresponding module was not added. The specific experimental results are shown in the table. The comparison of the results of the ablation experiments is shown in **Table 5**.

**Table 5:** Comparison of ablation experiment effects.

Number	Feature Enhancement Module	Feature Fusion Module	Attention Mechanism	mAP/%
1				77.81
2	√			78.61
3		√		77.57
4			√	79.29
5		√	√	79.14
6	√	√		78.76
7	√		√	79.91
8	√	√	√	79.39

The study’s results on the effectiveness of the feature enhancement module, feature fusion module, and attention mechanism SENet show that the mAP of the basic network on the diseased pine tree dataset is 77.81%. After adding the feature enhancement module, the mAP increased to 78.61%, resulting in a 0.8% improvement. The reason is that introducing the feature enhancement module can enhance the weight information of the target object and extract features more comprehensively and accurately. After adding the attention mechanism to the primary network, the mAP increased to 79.29%, resulting in a 1.48% improvement. As shown by the results of experiments 1 and 3, not all modules can improve the detection performance of the model. The mAP of the test set fell after adding the feature fusion module, indicating that the feature fusion module’s results were unstable and unsuitable for implementation in the YOLO v4 network. The mAP climbed to 79.91% after adding the feature enhancement module and attention mechanism to the original YOLO v4 network, representing a 2.1% improvement. The combination of the feature improvement module and the attention mechanism SENet was chosen to be the best network model after screening. Thus, added the SENet attention mechanism and the feature improvement module after the last three feature layers of the YOLO v4 backbone feature network, the accuracy of YOLO v4 disease tree detection has been improved 2.1%. The improvement of detection performance is related to the feature extraction ability of the feature enhancement module. The feature enhancement module is self-designed, which can adapt to different lighting changes.

### 4.2 Feature visualization analysis

**Figure 11:**
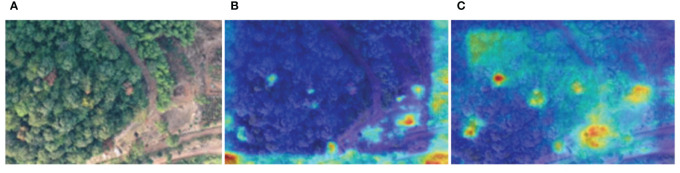
Thermal diagram before and after embedding the improved module. **(A)** Network Input Diagram. **(B)** The diagram before the improvement module is embedded. **(C)** The diagram after the improvement module is embedded.

The Gradient-weighted Class Activation Mapping (Grad-CAM) tool was used to analyze the feature extraction process of the network, extract heat maps after embedding the improvement modules, and analyze the impact of the improvement modules on target feature extraction. The brightest point at the center is the position of the center point, and the closer the position is to the vital point of the target, the larger the activation function value (**Figure 11**). The darker the color of the center point, the more obvious the feature. Before embedding the improvement modules, the YOLO v4 network randomly extracted the features of diseased trees and did not pay enough attention to the features of the diseased tree location. After embedding the improvement modules, the critical feature channels accounted for a more significant proportion, the network obtained a larger receptive field, and the improved YOLO v4 network could more effectively extract the feature information of diseased trees, making it easier to distinguish the location of diseased trees from the image. The improved YOLO v4 model performs better in detecting diseased trees, not only recognizing a larger number of diseased trees, but also improving the model’s ability to recognize green backgrounds as yellow diseased trees. The improved YOLO v4 model can extract more feature information about disease trees and improve the detection performance of disease trees under complex lighting conditions. In order to better achieve lightweight deployment of models, future research focuses on reducing model volume and improving detection speed while minimizing model accuracy loss.

### 4.3 Visualization of prediction results

**Figure 12:**
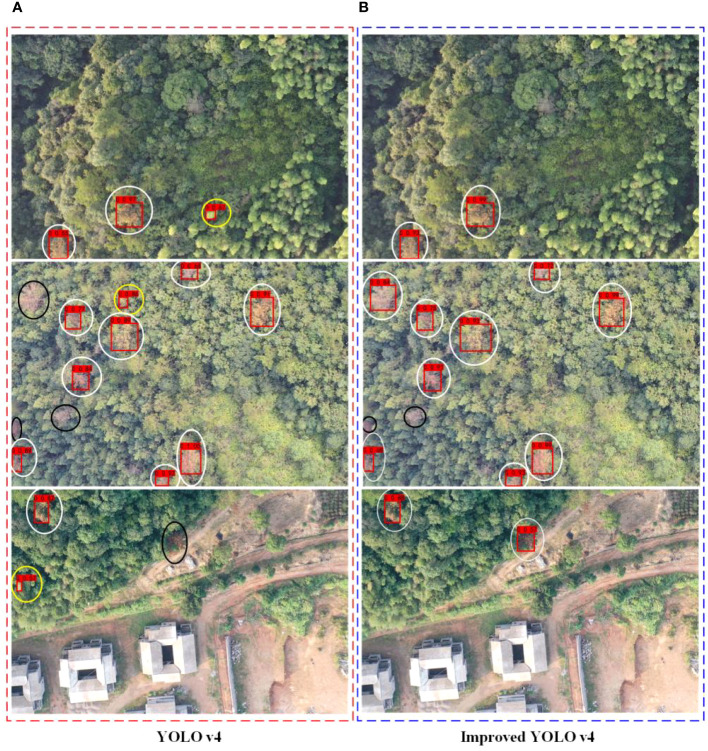
Remote sensing image recognition results under strong light environment. * white circles indicate correct detections, black circles indicate missed detections, yellow circles indicate misdetections. **(A)** YOLO v4 detection results **(B)** Improved YOLO v4 detection results.

The test set images were used to analyze and evaluate the results of diseased tree recognition. A total of 515 test set images were selected to evaluate the model’s prediction results, and the prediction results of two models in robust light environments are shown (**Figure 12**).

It can be seen that after the model was improved, it could detect the specific location of the diseased tree, and the confidence values were all increased (**Figure 12B**). In the predicted images, there were fifteen diseased trees of different colors with strong light, and some of the diseased tree crowns had small contours and colors similar to those of surrounding trees, as well as overlapping crowns. In this complex image background, both models could identify the location of the diseased trees accurately. Among them, the YOLO v4 model identified ten diseased trees, and three were not correctly identified, with false positives (**Figure 12A**). After adding the channel attention mechanism SENet and feature enhancement module, the improved YOLO v4 model correctly identified thirteen diseased trees, three more than the YOLO v4 model. The reason why the YOLO v4 model failed to detect the one missed diseased tree correctly may be due to the obstruction of other healthy trees in the crown, which affected the feature extraction of the model.

### 4.4 Comparative experiments with other object detection models

To compare the comprehensive performance of the improved YOLO v4 model in this study, Single Shot Multibox Detector (SSD), Faster RCNN, YOLO v3, and YOLO v5 were compared, showing the effectiveness of the model in detecting diseased pine trees, as shown in **Table 6**.

**Table 6:** Experimental comparison results of different models.

Models	mAP/%	Recall/%	Precision/%	Params/M
SSD	11.71	10.06	64.67	26.285
Faster RCNN	17.42	25.8	21.42	28.275
YOLO v3	25.23	21.95	84.00	61.949
Improved YOLO v4	79.91	67.15	86.36	256.82
YOLO v5	78.69	69.98	81.63	7.022

The improved YOLO v4 model has the highest parameters, which are increased by 230.535 M, 228.545 M, and 194.871 M compared to SSD, Faster RCNN, and YOLO v3, respectively. This is due to the addition of the SENet module and feature enhancement module to the YOLO v4 network.

Moreover, the improved YOLO v4 model has the highest mAP, which is increased by 68.2%, 62.49%, 54.68%, and 1.22% compared to SSD, Faster RCNN, YOLO v3, and YOLO v5, respectively. The model’s precision is also the highest, which has increased by 21.69%, 64.94%, 2.36%, and 4.73% compared to SSD, Faster RCNN, YOLO v3, and YOLO v5, respectively. Although, the improved YOLO v4 model has the highest parameters and requires more computation, its performance is the best, as its mAP is 79.91%, the highest among the five models, indicating that the improved YOLO v4 model has higher detection accuracy. Therefore, the model improvement in this study is effective.

## 5 Conclusion and discussion

Since the changes in lighting conditions can lead to a decrease in image quality during unmanned aerial vehicle detection of pine wilt disease, this study used unmanned aerial vehicles to create a sample set of diseased trees at different time periods, making the deep learning model trained more generalizable and improving the performance of object recognition. The application of the YOLO v4 algorithm in the field of diseased tree object detection was studied, and the CSPDarknet53 network structure was used to complete the feature extraction process. In contrast, the feature pyramid network structure was used to enhance the feature extraction capability of the convolutional neural network. The mAP of the YOLO v4 model was 77.81%. By comparing experiments, the type of attention mechanism and its addition position in the YOLO v4 network were determined, and the detection effect was best when the attention mechanism module SENet was added before the feature pyramid network structure. The ablation experiment found that the optimal combination was the object detection model that combined the channel attention mechanism SENet and feature enhancement module. The mAP of the model was 79.91%, an increase of 2.1% after improvement, indicating that the channel attention mechanism SENet combined with feature enhancement module can effectively enhance the ability to recognize detection targets. Under the same conditions, the mAP of the improved YOLO v4 model was increased by 68.2%, 62.49%, 54.68%, and 1.22% compared to SSD, Faster RCNN, YOLO v3, and YOLO v5, respectively, indicating that the model can achieve high-precision detection of diseased trees caused by PWD under changing light conditions. In 2021, Wu estimated the power of the hyperspectral method, LiDAR and their combination to predict the infection stages of PWD using the random forest (RF) algorithm. The results showed that the combination of hyperspectral method and LiDAR had the best accuracies (Yu et al., 2021). The improved YOLO v4 model has a high recognition accuracy for diseased trees, which can achieve precise positioning and recognition of pine wilt disease trees under changing light conditions. This is critical in guiding the prevention and control of pine wilt disease.

The ablation experimental results have demonstrated the optimization effect of the improved module on the YOLOv4 detection network. Although the improved YOLOv4 algorithm performs well in the target detection task of diseased tree images captured by drones, there is still room for improvement in detection accuracy and speed. The current challenge is how to count the number of diseased trees in the image, which requires post-processing of the model but increases its complexity. Following that, there is a goal to do research on lightweight models and build software and hardware implementation of a real-time target detection system suited for drones to detect disease trees. Moreover, the system provides ideas for lychee disease detection in lychee gardens.

## Data availability statement

The original contributions presented in the study are included in the article/supplementary material. Further inquiries can be directed to the corresponding authors.

## Author contributions

ZZ: Conceptualization, Formal analysis, Investigation, Methodology, Writing – original draft, Writing – review & editing. CH: Project administration, Software, Visualization, Writing – original draft, Writing – review & editing. XW: Conceptualization, Investigation, Writing – original draft, Writing – review & editing. HL: Data curation, Formal analysis, Writing – original draft, Writing – review & editing. JL: Supervision, Validation, Writing – original draft, Writing – review & editing. JZ: Methodology, Resources, Visualization, Writing – original draft, Writing – review & editing. SS: Data curation, Formal analysis, Resources, Validation, Writing – original draft, Writing – review & editing. WW: Funding acquisition, Resources, Writing – original draft, Writing – review & editing.
